# A Thermolabile Aldolase A Mutant Causes Fever-Induced Recurrent Rhabdomyolysis without Hemolytic Anemia

**DOI:** 10.1371/journal.pgen.1004711

**Published:** 2014-11-13

**Authors:** Asmaa Mamoune, Michel Bahuau, Yamina Hamel, Valérie Serre, Michele Pelosi, Florence Habarou, Marie-Ange Nguyen Morel, Bertrand Boisson, Sabrina Vergnaud, Mai Thao Viou, Luc Nonnenmacher, Monique Piraud, Patrick Nusbaum, Joseph Vamecq, Norma Romero, Chris Ottolenghi, Jean-Laurent Casanova, Pascale de Lonlay

**Affiliations:** 1 INSERM U781, Institut Imagine des Maladies Génétiques, Université Paris Descartes et Centre de Référence des Maladies Héréditaires du Métabolisme, Hôpital Necker, AP-HP, Paris, France; 2 Département de Génétique, Hôpitaux Universitaires Henri-Mondor, Créteil, AP-HP, France; 3 "Mitochondria, Metals and Oxidative Stress" group, Jacques Monod Institute, UMR7592 CNRS, Paris Diderot University, Paris, France; 4 Metabolic biochemistry and INSERM U1124, University Paris Descartes, Hospital Necker Enfants Malades, Paris, France; 5 Clinique Universitaire de Pédiatrie, Hôpital couple enfant, CHU de Grenoble, France; 6 St. Giles Laboratory of Human Genetics of Infectious Diseases, Rockefeller Branch, The Rockefeller University, New York, New York, United States of America; Unité Institut National de la Santé et de la Recherche Médicale U980, Laboratory of Human Genetics of Infectious Diseases, Imagine Institute; and Pediatric Hematology-Immunology Unit, Necker Hospital for Sick Children, University Paris Descartes, Paris, France; 7 Département de Biochimie, Toxicologie et Pharmacologie, CHU de Grenoble, Centre de Référence Rhône-Alpes des Maladies NeuroMusculaires, Grenoble, France; 8 Université Pierre et Marie Curie, UM 76, INSERM U974, CNRS UMR 7215, Institut de Myologie, GHU Pitié-Salpêtrière, AP-HP, Centre de Référence des Maladies Neuromusculaires, Paris, France; 9 Laboratoire Maladies Héréditaires du Métabolisme, Centre de Biologie et Pathologie Est, Hospices Civils de Lyon, Lyon, France; 10 Banque de Cellules, Hôpital Cochin, APHP, Paris, France; 11 INSERM et Laboratoire de Biochimie et Biologie Moléculaire, HMNO, CBP, CHRU Lille, Lille, France; 12 Howard Hughes Medical Institute, New York, New York, United States of America; University of Oxford, United Kingdom

## Abstract

Aldolase A deficiency has been reported as a rare cause of hemolytic anemia occasionally associated with myopathy. We identified a deleterious homozygous mutation in the *ALDOA* gene in 3 siblings with episodic rhabdomyolysis without hemolytic anemia. Myoglobinuria was always triggered by febrile illnesses. We show that the underlying mechanism involves an exacerbation of aldolase A deficiency at high temperatures that affected myoblasts but not erythrocytes. The aldolase A deficiency was rescued by arginine supplementation *in vitro* but not by glycerol, betaine or benzylhydantoin, three other known chaperones, suggesting that arginine-mediated rescue operated by a mechanism other than protein chaperoning. Lipid droplets accumulated in patient myoblasts relative to control and this was increased by cytokines, and reduced by dexamethasone. Our results expand the clinical spectrum of aldolase A deficiency to isolated temperature-dependent rhabdomyolysis, and suggest that thermolability may be tissue specific. We also propose a treatment for this severe disease.

**Abstract Summary**

Using recent technical advances involving exome analysis, we identified a new missense mutation in the *ALDOA* gene, encoding a key enzyme in the glycolytic pathway. The patients presented with severe recurrent rhabdomyolysis without hemolytic anemia. The decrease of aldolase A activity in myoblasts was enhanced at high temperature and could explain the fever-induced rhabdomyolysis. By contrast, enzyme thermolability was not found in erythrocytes, possibly accounting for the unusual clinical phenotype of the patients. Enzyme thermolability was rescued by arginine supplementation in vitro but not by other chaperone compounds.

## Introduction

Massive rhabdomyolysis is a life threatening condition and has been associated with mitochondrial fatty acid ß-oxidation defects (FAO) [1]–[3], *LPIN1* mutations [4]–[6], as well as, rarely, with mitochondrial respiratory chain (RC) deficiency, dystrophinopathies and inborn errors of glycogenolysis and glycolysis [3], [7]. Among inherited defects of glycolysis, isolated rhabdomyolysis is not an usual presentation. Because most metabolic mechanisms of rhabdomyolysis are triggered by fever, differential diagnoses include myositis and viral infections for non recurrent cases. Metabolic work-up focuses on plasma carnitine and acylcarnitine profiles, urinary organic acids analysis, then sequencing of *LPIN1* gene in young children. In older children, ischemic stress test can orient toward anaerobic glycolysis defects. In both young and older patients, skeletal muscle biopsy may be proposed for histological studies in cases of negative biochemical and molecular results. In spite of this wide range of investigations, the disease mechanism remains unknown in at least half of the recurrent cases [8].

In order to identify new etiologies of recurrent rhabdomyolysis in young children, we used exome sequencing in siblings suffering from severe episodes of rhabdomyolysis triggered by fever since age 2 months. This led us to identify a new phenotype of *ALDOA* mutations. The absence of hemolytic anemia was explained by tissue specific expression of protein thermolability. The occurrence of thermolability supports the contention that viral infections should remain a diagnosis of exclusion for rhabdomyolysis. Our results raise the possibility of medical therapy by arginine.

## Results

### Case report

**Figure 1:**
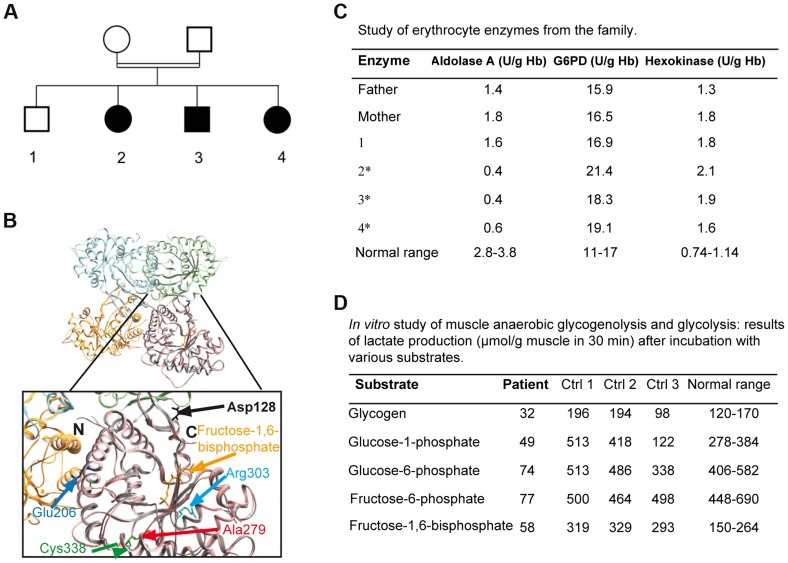
1A: Family tree showing the 3 affected children. 1B: Crystal structure of human muscle aldolase complexed with fructose 1,6-bisphosphate (isoenzyme A, PDB code 4ALD) superimposed with the tetrameric crystal structure of human brain aldolase (isoenzyme C, PDB code 1XFB), which is similar to the muscle isoenzyme. Chains A, B, C and D of isoenzyme C are shown in orange, light blue, light green and pink, respectively. Monomeric isoenzyme A is shown in grey and is superimposed on chain D of the tetrameric isoenzyme C. Fructose 1,6-bisphosphate co-crystallized with isoenzyme A is shown in yellow. The mutated residue described in this report (red arrow) and the mutated amino acids previously described are highlighted in the magnified structure. The structural and functional consequences of the mutations are described in Table 1. 1C: aldolase A, glucose-6-phosphate dehydrogenase (G6PD) and hexokinase activities in the erythrocytes of the parents, the healthy sibling and the 3 affected patients (*: patients 2, 3, 4). 1D: in vitro muscle study of anaerobic glycogenolysis and glycolysis (only patient 3); results of lactate production (µmol/g muscle in 30 minutes) after incubation with various substrates.

Three patients from a Moroccan consanguineous family (Figure 1A) suffered from recurrent episodes of rhabdomyolysis that required numerous hospitalizations from 2 months of age. These acute episodes were invariably triggered by febrile illnesses. The presenting symptoms were an inability to walk and myalgia. During the acute episodes, plasma creatine phosphokinase (CK) levels were variable, ranging from markedly elevated (peak levels: 180,000–450,000 U/L, N<150) with overt myoglobinuria to milder elevations (3,000 U/L). The following tests were normal: hemoglobin, hematocrit, mean corpuscular volume, plateletcount, reticulocyte count, bilirubin, haptoglobin, ferritin, Coombs' test, urea, creatinine, blood gasses, plasma lactate, carnitine, blood acylcarnitine profile, plasma amino acids, and urinary organic acids. Electromyography, brain MRI, abdominal ultrasonography, and echocardiography were also normal. CK levels ranged from normal (<150 U/L) to elevated (up to 1,800 U/L) in all 3 patients between acute episodes. The clinical examination and muscle tests performed 2 months after an episode of rhabdomyolysis were normal for each patient, at ages 9, 10, and 11 years respectively. Family history revealed neither chronic hemolytic anemia, nor episodes of jaundice or blood transfusions. Two patients suffered from learning disabilities and required a special school.

### Molecular studies

Exome sequencing analysis pointed to 10 candidate genes harboring homozygous mutations (Table S1). The ALDOA gene was considered because all 3 affected patients harbored the homozygous mutation c.839 C>T (p.Ala279Val, NM_000034), whereas the healthy sibling and the parents were heterozygous. This gene was located in a homozygous region revealed by homozygosity mapping, between the polymorphic markers D16S3022 and D16S323. Polyphen software predicted that this change, located in a sequence conserved between species (Figure S1), was “probably damaging”. The homozygous mutation p. Ala279Val was localized near the previously described Cys338residue involved in a hematological and muscle phenotype[9] close to a “hinge” region that is critical for a conformational change in the C-terminus (Figure 1B). Prediction from seven combined softwares as implemented in iSTABLE [10] was consistent with A279V destabilizing the protein. Therefore, this mutation is likely involved in maintaining the correct spatial conformation of the enzyme (see below) [9], [11].

### Morphological studies

All histological and cellular experiments were performed with the tissue and myoblasts from the same patient aged 10 (Patient 3, Figure 1C).

**Figure 2:**
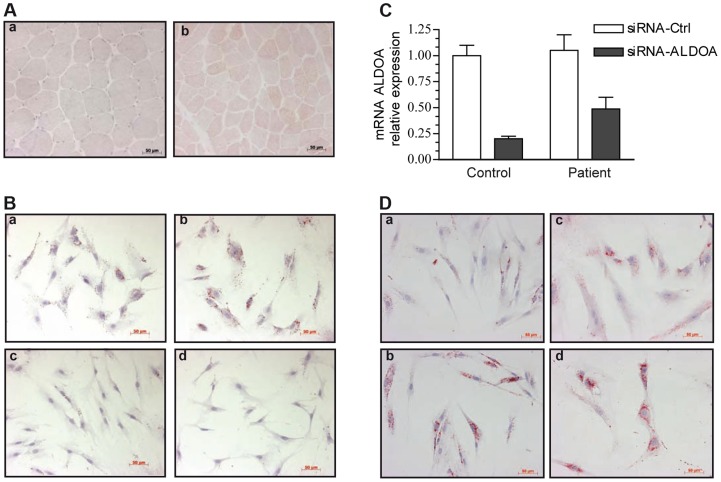
Oil-red-O staining of skeletal muscle and myoblasts from our patient and a control. Images were taken with x20 magnification. 2A: Transverse cross-section of a left deltoid muscle biopsy of the patient shows the presence of numerous LDs, mainly in type 1 fibers, a: control, b: patient. 2B: Cytological oil-Red-O analysis of the patient myoblasts cultivated under basal (a) or pro-inflammatory conditions (TNFα+IL-1β) (b). LDs appear as red circular vacuoles in the cytoplasm. Treatment with dexamethasone alone (d) or combined with TNFα+IL-1β (c) reversed the LD phenotype. 2C: Relative knockdown of aldolase A expression in control and patient myoblasts. 2D: representative oil-red-O staining of mock-transfected control (a) and patient (c) cells, or aldolase A-siRNA-transfected control (b)and patient (d) myoblasts.

Microscopy showed an excessive lipid droplet (LD) accumulation, visualized with oil-red-O staining (Figure 2A), in the muscle biopsy from the patient (a) compared with control biopsies (b). However, the muscle tissue of the patient showed a well-preserved fascicular architecture with normal type 1 and type 2 fibers. In addition, the cytochrome *c*-oxidase and phosphorylase staining patterns were similar to controls.

Representative cultures of the patient under basal (a) or pro-inflammatory conditions (b) are shown in Figure 2B. Oil-red-O staining of the neutral lipids revealed massive accumulation of LDs after 24 hours of TNFα+IL-1ß stimulation (b), whereas no change in the size or the number of the LDs was observed at 40°C (Figure S2). In contrast, control myoblasts exhibited no LDs under basal condition and a moderate accumulation after TNFα+IL-1ß stimulation (Figure S3). Co-treatment with anti-inflammatory agents and particularly treatment with the synthetic glucocorticoid dexamethasone combined with TNFα+IL-1ß (c) or alone (d), dramatically decreased the number of LDs in patient myoblasts.

The effect of the *ALDOA* knockdown on LD accumulation was investigated in order to test if muscle abnormalities seen in patients result from *ALDOA* decrease. Transfection with a siRNA that knocked down *ALDOA* expression (as confirmed by RT-PCR, Figure 2C), induced a 48% increase in the number of LDs in the control myoblasts (Figure 2Da,b) and a 5.5% increase in the patient cells (Figure 2Dc,d).

### Biochemical studies

ALDOA activity was dramatically decreased in patient erythrocytes (0.4 to 0.6 U/g Hb; control 4.6) (Figure 1C) and frozen skeletal muscle (55 nmol/h/mg; normal 581-5188) though to a lesser degree in myoblasts (1.08±0.4 U/µg protein; normal 2.4±0.025). Activity was approximately half the normal in the heterozygote (Figure 1C), supporting the pathogenic nature of the c.839 C>T mutation and the recessive inheritance of the disease. Studies of muscle anaerobic glycogenolysis and glycolysis *in vitro* revealed reduced lactate production, consistent with dysfunctional glycolysis (Figure 1D). The glucose-6-phosphate dehydrogenase and hexokinase activities were normal or elevated in all affected patients' erythrocytes (Figure 1C). Carbons from stable isotope labeled glutamine were incorporated to a lesser degree into Krebs cycle intermediates in the patient myoblasts relative to controls, consistent with *ALDOA* deficiency leading to decreased glycolytic flux (Figure S4).

**Figure 3:**
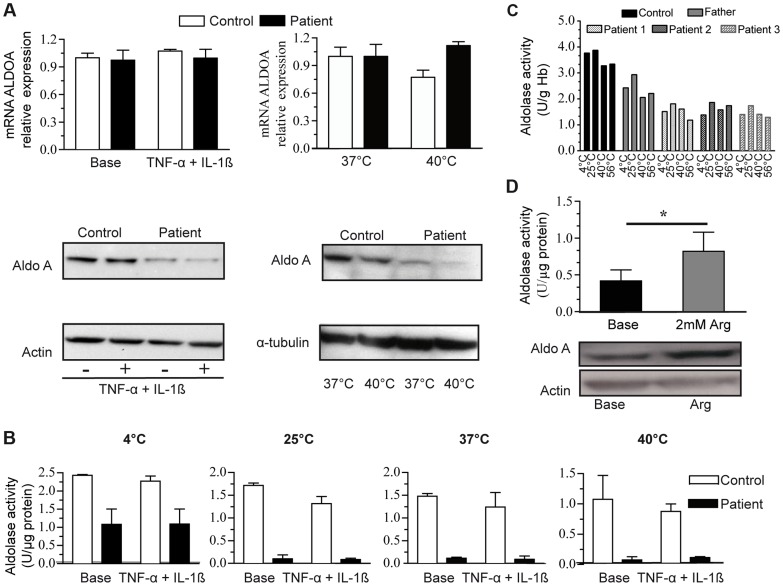
*ALDOA* expression and activity. 3A:*ALDOA* mRNA expression in control myoblasts (C, white bars) and the patient myoblasts (P, grey bars) under basal conditions, with TNFα+Ilβ treatment (left) or at a high temperature (right, 40°C); Aldolase A protein levels (lower panel) under basal conditions, with TNFα+Ilβ treatment or at a high temperature. 3B: Aldolase A activity in control and the patients' myoblasts under the same conditions: basal conditions, TNFα+Ilβ treatment and at different temperatures. The results are shown as the mean value ±SD from 3 independent experiments. 3C: Aldolase A activity in control and patients erythrocytes under basal conditions and at different temperatures. The results are shown as the mean value of two independent experiments. 3D: Aldolase A activity (upper) and protein level (below) in the patient myoblasts under basal condition and after arginine (Arg) treatment.*: p<0,05).

We studied whether temperature or pro-inflammatory cytokines affected *ALDOA* expression. To this end, the patient and the control myoblasts were cultured at 37 or 40°C, under basal conditions or with the combination of TNFα+IL-1β. Myoblasts from the patient and the controls responded to pro-inflammatory stress by significant secretion of IL6, peaking at approximately 24 hours of TNFα+IL-1ß stimulation (20-fold). The level of *ALDOA* mRNA was unchanged in patient myoblasts after exposure to TNFα+IL-1β or 40°C (Figure 3A upper panel), whereas the corresponding protein level was reduced in patient myoblasts in basal conditions (0.6±0.09; control: 1.3±0.17) and was further abated at 40°C (barely detectable) compared to control (0.8±0.16) (Figure 3A, lower panel). In contrast, TNFα+IL-1β treatment did not affect the protein level (1.2±0.11 to 0.97±0.11 in control and 0.7±0.09 to 0.8±0.13 in patient myoblasts). Accordingly, aldolase activity in the patient myoblasts dramatically decreased after incubation from 25°C, 37°C through 40°C (residual activity 10% and 5% respectively), and to a lesser degree in the control myoblasts (residual activity 61% at 37°C and 43% at 40°C) (Figure 3B). TNFα+IL-1β treatment did not change the level of activity in the patient or the control myoblasts (Figure 3B). Interestingly, aldolase A activity in erythrocytes from the three patients was not modified by high temperature (Figure 3C). These results suggest that the mutant enzyme might be differentially destabilized in distinct tissues, i.e., only in myoblasts and not in erythrocytes.

Arginine supplementation significantly enhanced aldolase A activity in patient myoblasts compared to untreated cells (Figure 3D, upper panel); this effect may be explained by an increase of the protein level (Figure 3D, lower panel). By contrast, glycerol, benzylhydantoin and betaine did not modify aldolase activity and protein (Table S2), suggesting that arginine-mediated rescue did not operate as a protein chaperone. Arginine did not contribute carbons to Krebs cycle intermediates in 6-hr stable isotope labeling experiments in myoblasts (Figure S4), thus indicating that arginine did not represent a major source of compensatory fuel for energy production.

The respiratory chain activities were increased in the frozen skeletal muscle compared to control and similar in the myoblasts of the patient and the control (Table S3). Similarly, fatty acid oxidation (FAO) results were normal in patient myoblasts compared to control and were not affected by pro-inflammatory conditions (Table S4).

## Discussion

Massive rhabdomyolysis is a life-threatening situation, yet a molecular mechanism is found in only half of the recurrent cases. Because glycolysis is the most important source of energy in erythrocytes and in some types of skeletal muscle fibers, inherited defects of glycolysis can cause hemolytic anemia or the combination of hemolytic anemia, neurologic abnormalities, and myopathy [12]. By exome sequencing and homozygosity mapping, we identified a new phenotype associated with aldolase A deficiency. The phenotype affects skeletal muscle with no hemolytic symptoms, while mild learning disabilities were identified in 2 of the 3 siblings.

Only 5 patients with aldolase A deficiency have been reported so far (Table 1) [9], [13]–[16]. All presented with non-spherocytic hemolytic anemia (OMIM #103850), a few had mental retardation [13], [15] and 2 also displayed rhabdomyolysis leading to death [9], [13]. Because of the involvement of *ALDOA* in skeletal tissue function and the observation of muscle symptoms in 2 previous cases with hemolytic anemia, we inferred that *ALDOA* mutations could be responsible for rhabdomyolysis in our family. In agreement with this, we showed the effect of the *ALDOA* knockdown on LD accumulation in patient myoblasts, and that they were enhanced by *ALDOA* siRNA in non-inflammatory conditions compared to control. LD accumulation was further enhanced in inflammatory conditions, consistent with the fact that intracellular accumulation of lipids is a common feature of proinflammatory stress [17], [18]. LD are dynamic organelles and provide key storage compartments that participate in the response to increased energetic demand [19] and in the regulation of cellular lipid metabolism, thus being involved in the delicate balance between triacylglycerol deposition and mobilization. LD accumulation as frequently observed in energetic diseases could result from a metabolic adaptation of the affected cells.

**Table 1:** Reported cases of Aldolase A deficiency with the described mutations.

Ethnicity	Consanguinity	Mutation	Clinical description			Aldolase A activity (U/gHb)		Consequences	Ref.
			Hemolytic anemia	Myopathy	Mental retardation	Patient	Control		
Japanese	Probable	p.Asp128Gly	Yes	No	No	0,12	2,99	AA in the subunit interface essential for the tetrameric structure.Thermolability	Miwa et al,1981; Kishi et al,1987
German	No	p.Glu206Lys	Yes	Yes	No	0.3	7.9	AA in the subunit interface essential for the tetrameric structure	Kreuder et al, 1996
Sicilian	No	p.Arg303X p.Cys338Tyr	Yes	Yes	No	0.3	1.3-2.8*	AA involved in maintaining the correct spatial conformation	Yao et al, 2004
Moroccan	Yes	p.Ala279Val	No	Yes	Yes	0.4	4.6	AA near a critical “hinge” region determining the position of the flexible C-terminal region required for correct activity	Current report

*: normal range. In the first case reported by Beutler et al in 1973 with no described mutation, the red cell aldolase activity was 16% of normal mean.

We were puzzled to find no evidence of hemolytic anemia in our patients although residual enzyme activity was 9% in erythrocytes and 45% in skeletal myoblasts. This may be related to the greater residual enzyme activity observed in our patients (approximately 10%) compared to previously reported cases of *ALDOA* deficiency (approximately 5%). Also, the hematological investigation was performed outside of an acute episode of decompensation. Of note, alternative explanations cannot be ruled out, including variable expressivity (degree of metabolic stress) or a selective resistance to thermolability in erythrocytes (cell type specific protein-protein interactions).

Because aldolase A is physiologically active as an homotetramer, the integrity of the quaternary structure was suggested to confer thermal stability to the enzyme [20] and was found to be vulnerable in erythrocytes [11], [16] as well as according to iSTABLE prediction [10]. However, thermolability of aldolase A was found only in patient myoblasts, not in erythrocytes, and it was observed at lower temperature than previously described [11], [13], [14], [16]. Red blood cells contribute to about 40% of the blood volume and are the first cellular structures to respond to increased reactive oxygen species (ROS) activity [21]. Because they do not have nucleus, they rely on pre-existing proteins for protection against ROS damage [22] and possess an extensive array of antioxidants [23]. Also, they develop a system of defenses that represent an excellent example of redox balance maintenance [24]. Interestingly, we showed that protein and catalytic breakdown can be reversed by arginine but not by glycerol, benzylhydantoin and betaine which all might act as a chaperone as recently proposed for unrelated metabolic disorders [25]–[27]. Also, another mechanism may be proposed concerning the effective role of L-arginine on aldolase A activity, namely its antioxidant function via increased NO formation and reduced release of superoxide.

Wen et al. [28] described the role of the inflammasome complex and IL-1β [29] in the Warburg effect of aerobic glycolysis, which was recently shown to be promoted by lipopolysaccharides. [30] Conversely, glucose metabolism plays a crucial role in IL-1β transcription because high glucose boosts the production of IL-1 β in pancreatic beta cells. [31] However, myoblasts from the patient did not exhibit altered or abnormal IL-1β secretion. Moreover, the levels of most cytokines measured (see above) were undetectable or very low in patients' plasma. Similar results were obtained for healthy donors.

Due to LD accumulation in our patient muscle cells, consistent with findings in a previous patient [13], and because adenosine triphosphate (ATP) has several origins, we also examined FAO metabolism and OXPHOS. Moreover, pro-inflammatory cytokines are known to down regulate lipid metabolism. [32] However, FAO and OXPHOS metabolism were normal compared to control myoblasts, and no change in patient myoblasts was observed after TNFα+IL-1β treatment. LD accumulation may result from anaplerotic dysfunction of the Krebs cycle. [33] Consistent with this possibility, we found reduced incorporation of carbon from stable isotope labeled glutamine (Figure S4). A number of secondary events may also play a role in muscle pain such as lipotoxicity [34] and osmotic and ionic modifications with consequences for ionic exchange. [35] The role of ALDOA as a scaffold protein that coordinates actin and microtubule networks can also be speculated to participate in the biogenesis of lipid droplet. [36], [37] [38] Aldolase A has been shown to bind to vacuolar-type H^+^-ATPase (V-ATPase) with ARNO (an ADP-ribosylation factor guanine nucleotide exchange factor). [39] Interestingly, ARNO has been recently found to restore/promote lipid droplet formation. [40] Therefore, attractive mechanisms may stem from putative aldolase-ARNO interacting properties.

In conclusion, aldolase A deficiency is a rare cause of severe myoglobinuria in early childhood, as a consequence of impaired generation of ATP to fuel muscle metabolism. Our study points to the crucial role of fever as the trigger of rhabdomyolysis in our patients. High temperature and a combination of pro-inflammatory cytokines, utilized to mimic inflammatory conditions, led to decreased aldolase A activity and LD accumulation, respectively, in both the patient myoblasts and to a lesser extent, in the control myoblasts.

Thermolability selectively found in myoblasts and not in erythrocytes of the patients plays a crucial role in the pathophysiology of this disease. Finally, we showed that arginine may be a useful therapy that enhances the enzymatic activity in patient cells, probably more by another role, i.e. its antioxidant effect than by a chaperone role, whereas inflammatory conditions enhanced LD accumulation and, therefore, lipotoxicity.

## Materials and Methods

### Molecular studies

To identify the causative mutations for rhabdomyolysis in this family, exome sequencing was performed in 1 child using previously described methods. [41] All variants were annotated using an in-house-developed annotation software system. The variants were classified as previously unidentified when they were absent from the control populations and from all datasets, including dbSNP129, the 1000 Genomes Project, and in-house exome data.

The coding regions and flanking splice sites of the *ALDOA* (NM_000034) gene were sequenced using genomic DNA prepared from leukocytes.

Real-time quantitative PCR (RT-qPCR) assays were performed using cDNAs from myoblasts. All experiments were conducted in triplicate using an ABI PRISM 7300 Sequence Detection System instrument with SYBR Green fluorescence dye (Applied Biosystems).

The protein concentrations in myoblasts were determined using the Bradford method (Sigma).Forty micrograms of protein were separated using denaturing PAGE and transferred to PVDF membranes. After probing with the suitable antibodies (ALDOA (sc-12059, Santa Cruz), α-tubulin (T9026, Sigma) or β−actin (sc-81178, Santa Cruz)), the signals were detected using the ECL kit (GE Healthcare).

Swiss-Pdb Viewer 3.7 (http://www.expasy.org/spdbv) was used to analyze the crystal structure of the human muscle fructose 1,6-bisphosphate aldolase complex (PDB code 4ALD). Prediction of protein stability changes was obtained from seven combined softwares as implemented in iSTABLE [10].

The ethics committee of the Necker Hospital approved the research proposal. Informed consent was obtained from the siblings' parents.

### Morphologic and biochemical studies

Histological studies were performed on a skeletal muscle biopsy and myoblasts obtained from Patient3 left deltoid muscle (Figure 1A) as previously described. [42] Samples from 2age- and sex-matched controls were obtained. Myoblasts were subjected to various stress conditions mimicking those believed to trigger the episodes of rhabdomyolysis, including high temperature (40°C) [13] and pro-inflammatory cytokines TNFα+IL-1ß(10 ng/mlfor 24 hours; all from R&D Systems). [43]To evaluate the response of myoblasts to these stimuli,IL6/IL8 release into the culture medium was measured using an immunoradiometric assay kit (Immunotopics). [44] An inflammatory cytokine kit was used to determine the contents of 10 cytokines (BD, Bioscience) in blood plasma. The cytokine inhibitors anakinra (inhibitor of the IL-1β receptor, 1 µg/mL) and ab9635 (inhibitor of TNFα, 1 µg/mL), or the synthetic glucocorticoid dexamethasone (0.2 µM) were added to the culture medium 1 hour or 12 hours, respectively, prior to the 24 hour-incubation with TNFα+IL-1β.

The enzymatic activities in erythrocytes and myoblasts were determined according to the methods of the International Committee for Standardization in Hematology. [45] To study the effect of temperature, myoblast extracts and erythrocytes were incubated for 30 minutes at 4°C, 25°C, 37°C, 39°C,or 40°C before enzyme assay. In the presence of suitable cofactors (including ATP and NAD^+^) and substrates for multiple enzymes involved in glycolysis (Figure1D), we measured L-lactate formation after incubation of frozen homogenized muscle tissue at 37°C for 30 min under nitrogen gas according to a published protocol. [46]

Mitochondrial OXPHOS activities of the myoblasts were evaluated as previously described. [47] Fatty-acid oxidation (FAO) measurements were performed through the assay of deuterated C_2_ to C_16_ acylcarnitines generated by incubation of intact myoblasts with a pentadeuterated C16 fatty acid ([16-^2^H_3_, 15-^2^H_2_]-palmitate) according to a procedure used for the detection of β-oxidation defects. [48]

RNA-silencing (siRNA) experiments were performed on myoblasts using the jePRIME transfection reagent (Polyplus) according to the supplier's recommendations and 25 nM of siRNA targeting human AldoA(M-010375,Dharmacon). The non-targeting siRNA #2 (Dharmacon) was used as a negative control.

The effect of arginine supplementation was investigated in patients' myoblasts incubated with 2 mM of L-arginine hydrochloride (Sigma) for 10 days. Three other chaperons (sigma) were also tested, glycerol (100 mM) [25] for 10 days, betaine (10 and 50 mM) [26], [27] and benzylhydantoin (130 µM) for 3 days. Uniformly stable isotope labeled (U-^13^C_5_) glutamine or (U-^13^C_6_) arginine (Eurisotop, Saint-Aubin, France) were provided to myoblasts (1 mM) in the presence of glucose (2.5 mM) and incubated without serum for 6 hours. Krebs cycle intermediates and their isotopomers were measured by gas chromatography mass spectrometry (Scion TQ, Brüker) using standard derivation by BSTFA [N,O-Bis(trimethylsilyl)trifluoroacetamide] and 1% trimethylchlorosilane.

## Supporting Information

Figure S1 Aldolase A amino acid sequence alignment. The partial AldoA patient's sequence was aligned to orthologs from mouse (P05064), rabbit (P00883), zebra-fish (AAN04476.1). Human Aldolase A (P04075).
(TIFF)

Figure S2 Oil-red-O staining of myoblasts from our patient. No change in the size or the number of the LDs was observed in patient myoblasts at 40°C.
(TIFF)

Figure S3 Oil-red-O staining of myoblasts from a control. Control myoblasts exhibited no LDs under basal condition and a moderate accumulation after TNFα+IL-1ß stimulation.
(TIFF)

Figure S4 Stable isotope labeling experiments showing reduced ratios of labeled to natural isotope Krebs cycle intermediates (citrate, succinate, fumarate and malate) in patient myoblasts treated with uniformly labeled 1 mM glutamine (GLN*) relative controls, thus consistent with partial dysfunction of central energy metabolism in ALDOA deficient fibroblasts. No incorporation of arginine (ARG*) was observed in either patient or control myoblasts, indicating no major contribution of arginine to energy production. Bottom right: schematic of “central metabolism” as discussed in the text related to glutamine and arginine. Dotted lines indicate that one or more enzymatic steps are omitted for the sake of simplicity”.
(TIFF)

Table S1 Candidate genes with exome analysis.
(PDF)

Table S2 Aldolase A activity in patient myoblasts treated with glycerol, betaine and benzylhydantoin.
(PDF)

Table S3 Increased respiratory chain activities in skeletal muscle of the patient and similar respiratory chain activities in myoblasts of the patient and control, in basal condition and in pro-inflammatory conditions (T+I). T: TNF-α; I: IL-1β.
(PDF)

Table S4 Normal fatty acid oxidation in patient myoblasts compared to control, in basal and pro-inflammatory conditions (TNF-α + IL-1β).
(PDF)
