# Supplementary figures and images for: A Thermolabile Aldolase A Mutant Causes Fever-Induced Recurrent Rhabdomyolysis without Hemolytic Anemia
Source: PLoS Genet. 2014 Nov 13;10(11):e1004711. doi: 10.1371/journal.pgen.1004711 (PMC4230727; doi:10.1371/journal.pgen.1004711)

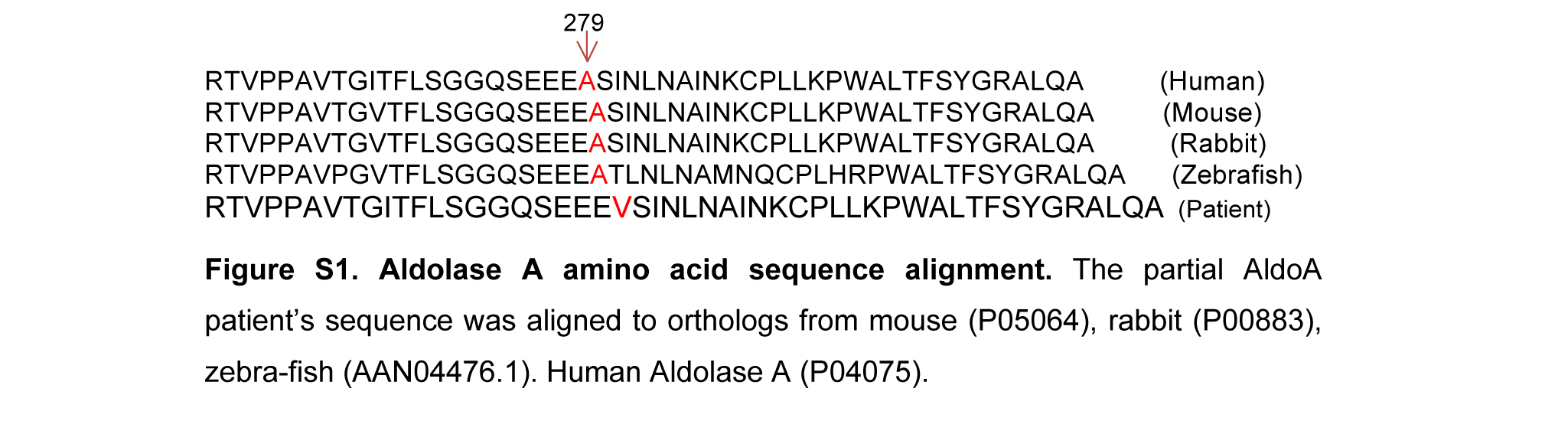

Supplement: Figure S1 — Aldolase A amino acid sequence alignment. The partial AldoA patient's sequence was aligned to orthologs from mouse (P05064), rabbit (P00883), zebra-fish (AAN04476.1). Human Aldolase A (P04075). (TIFF) [file pgen.1004711.s001.tiff]

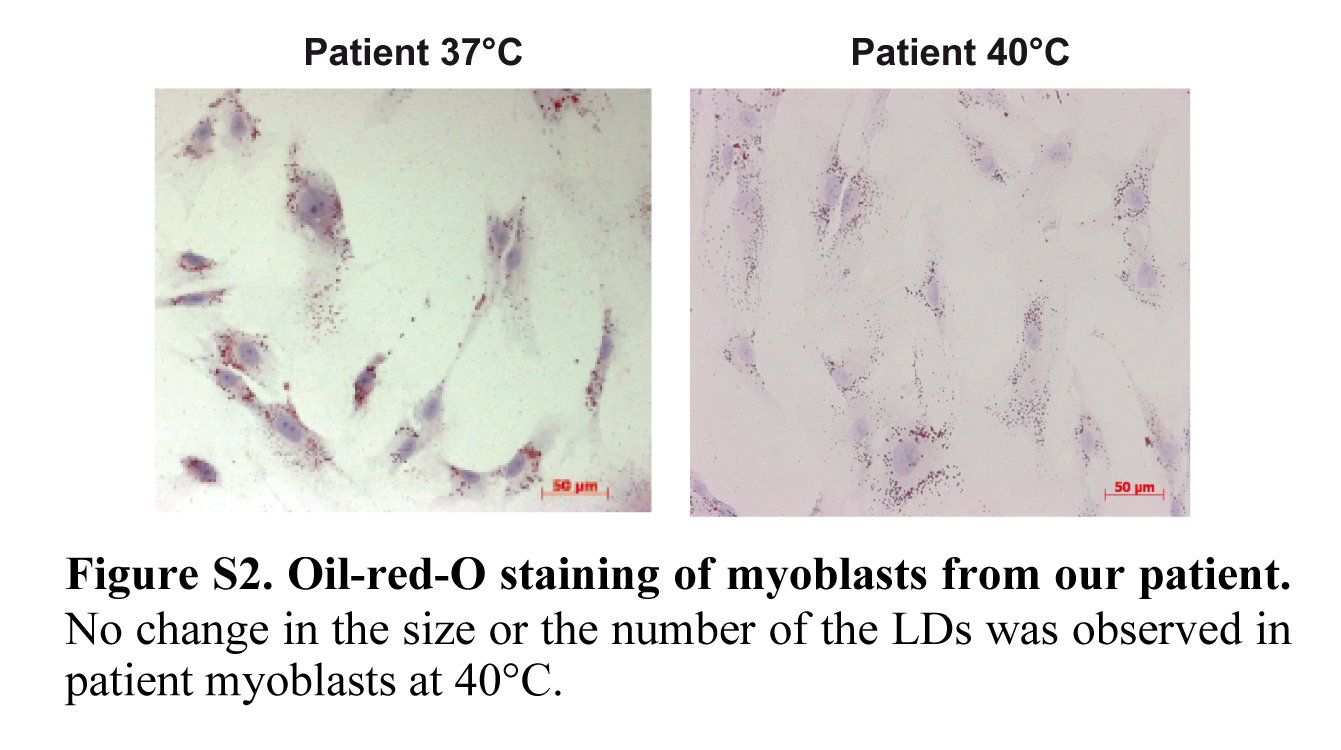

Supplement: Figure S2 — Oil-red-O staining of myoblasts from our patient. No change in the size or the number of the LDs was observed in patient myoblasts at 40°C. (TIFF) [file pgen.1004711.s002.tiff]

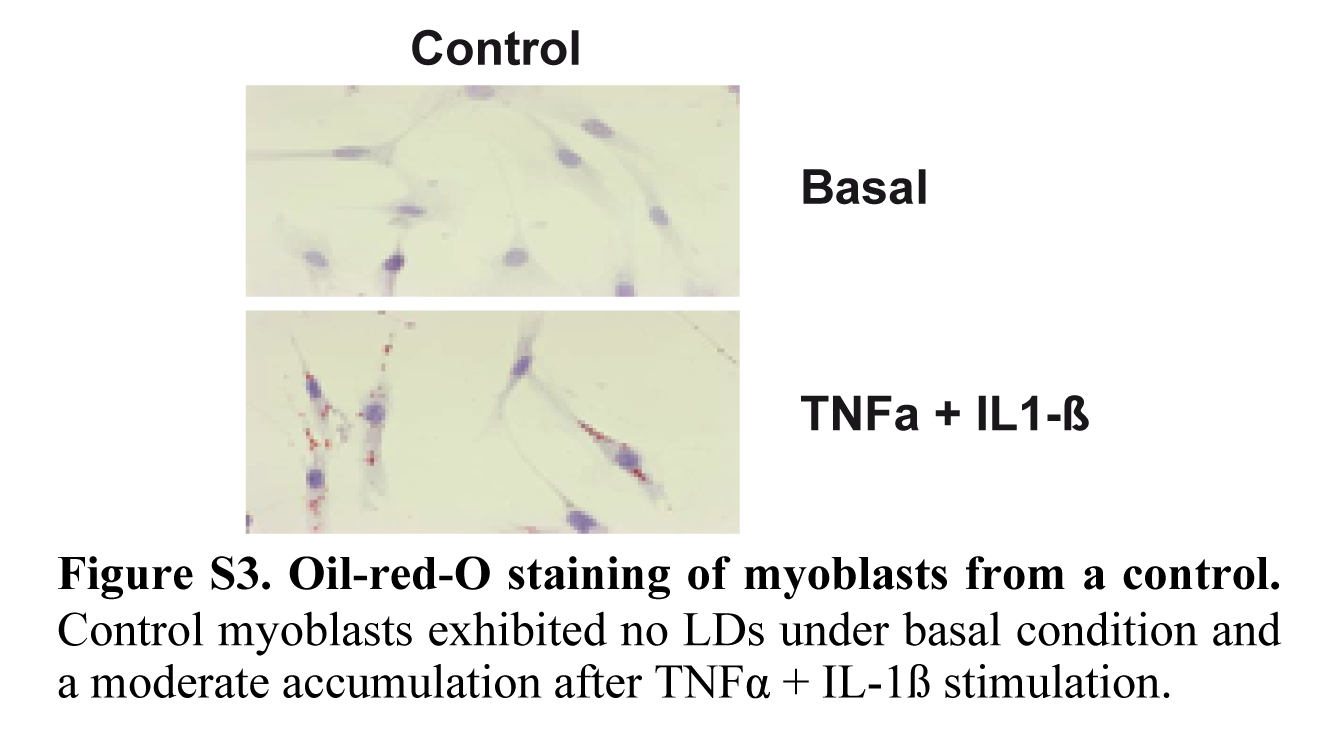

Supplement: Figure S3 — Oil-red-O staining of myoblasts from a control. Control myoblasts exhibited no LDs under basal condition and a moderate accumulation after TNFα+IL-1ß stimulation. (TIFF) [file pgen.1004711.s003.tiff]

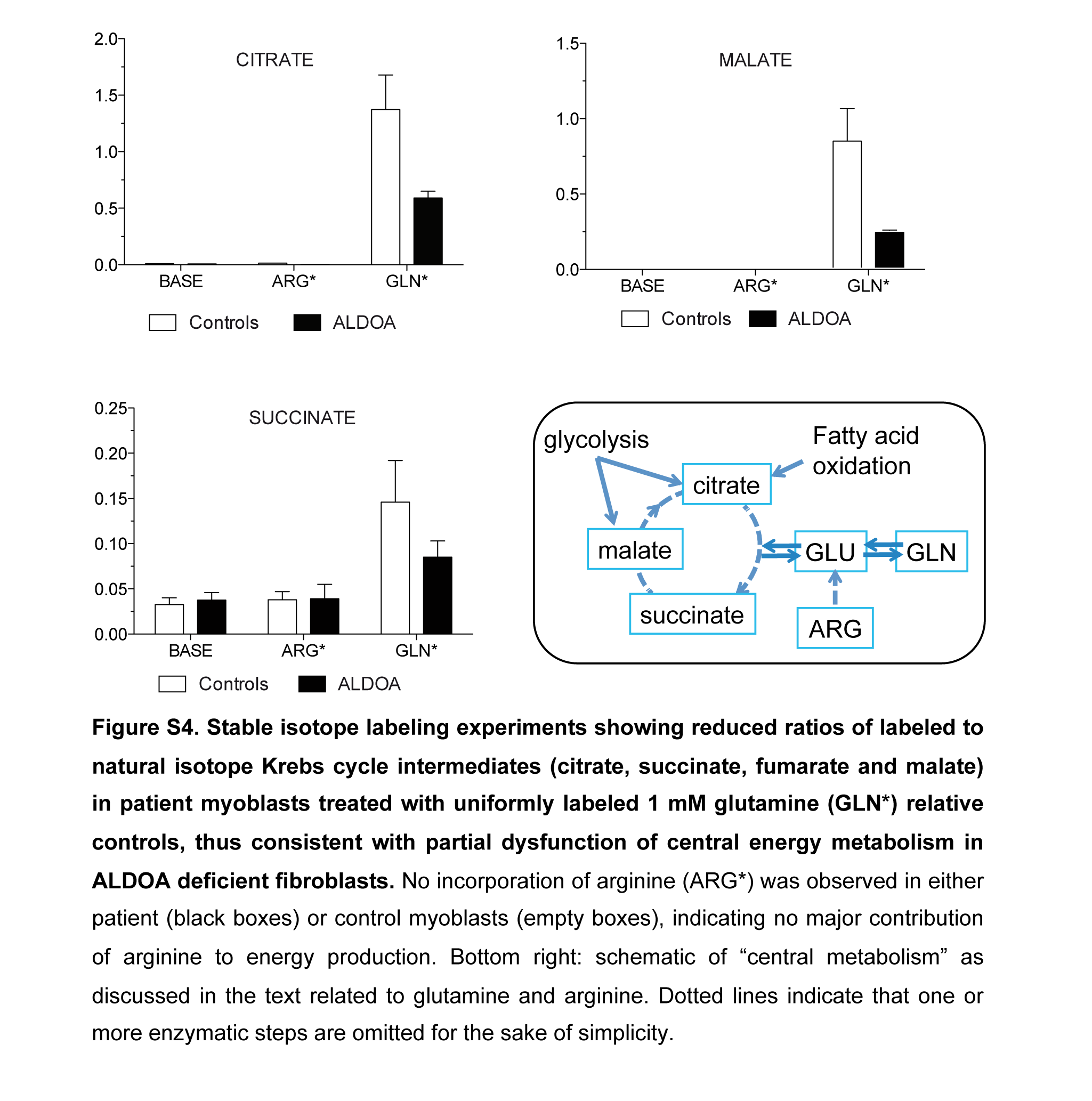

Supplement: Figure S4 — Stable isotope labeling experiments showing reduced ratios of labeled to natural isotope Krebs cycle intermediates (citrate, succinate, fumarate and malate) in patient myoblasts treated with uniformly labeled 1 mM glutamine (GLN*) relative controls, thus consistent with partial dysfunction of central energy metabolism in ALDOA deficient fibroblasts. No incorporation of arginine (ARG*) was observed in either patient or control myoblasts, indicating no major contribution of arginine to energy production. Bottom right: schematic of “central metabolism” as discussed in the text related to glutamine and arginine. Dotted lines indicate that one or more enzymatic steps are omitted for the sake of simplicity”. (TIFF) [file pgen.1004711.s004.tiff]
