# Supplementary material for: A Thermolabile Aldolase A Mutant Causes Fever-Induced Recurrent Rhabdomyolysis without Hemolytic Anemia
Source: PLoS Genet. 2014 Nov 13;10(11):e1004711. doi: 10.1371/journal.pgen.1004711 (PMC4230727; doi:10.1371/journal.pgen.1004711)
Supplement: Table S1 — Candidate genes with exome analysis. (PDF) [file pgen.1004711.s005.pdf]

Table S1. Candidate genes with exome analysis.

Gene	Chromosome	Description
NBPF10	1	neuroblastoma breakpoint family, member 10 [Source:HGNC Symbol;Acc:31992]
MLXIP	12	MLX interacting protein [Source:HGNC Symbol;Acc:17055]
CLIP1	12	CAP-GLY domain containing linker protein 1 [Source:HGNC Symbol;Acc:10461]
TDRD9	14	tudor domain containing 9 [Source:HGNC Symbol;Acc:20122]
TCF25	16	transcription factor 25 (basic helix-loop-helix) [Source:HGNC Symbol;Acc:29181]
ALDOA	16	
CCDC150	2	coiled-coil domain containing 150 [Source:HGNC Symbol;Acc:26834]
KCNJ6	21	potassium inwardly-rectifying channel, subfamily J, member 6 [Source:HGNC Symbol;Acc:6267]
CRIPAK	4	cysteine-rich PAK1 inhibitor [Source:HGNC Symbol;Acc:26619]
DCHS2	4	dachsous 2 (Drosophila) [Source:HGNC Symbol;Acc:23111]
