# Supplementary material for: A Thermolabile Aldolase A Mutant Causes Fever-Induced Recurrent Rhabdomyolysis without Hemolytic Anemia
Source: PLoS Genet. 2014 Nov 13;10(11):e1004711. doi: 10.1371/journal.pgen.1004711 (PMC4230727; doi:10.1371/journal.pgen.1004711)
Supplement: Table S2 — Aldolase A activity in patient myoblasts treated with glycerol, betaine and benzylhydantoin. (PDF) [file pgen.1004711.s006.pdf]

Table S2. Aldolase A activity in patient myoblasts treated with Glycerol, betaine and Benzylhydantoin.

	AldoA (U/µg protein)±SD
Basal condition	1.08 ± 0,1
Glycerol 100 mM	1.01 ± 0,2
Betaine 10 nM	0.92 ± 0,2
Betaine 50 nM	0.91 ±0,1
Benzylhydantoin 130 µM	0.82
