# Supplementary material for: A Thermolabile Aldolase A Mutant Causes Fever-Induced Recurrent Rhabdomyolysis without Hemolytic Anemia
Source: PLoS Genet. 2014 Nov 13;10(11):e1004711. doi: 10.1371/journal.pgen.1004711 (PMC4230727; doi:10.1371/journal.pgen.1004711)
Supplement: Table S3 — Increased respiratory chain activities in skeletal muscle of the patient and similar respiratory chain activities in myoblasts of the patient and control, in basal condition and in pro-inflammatory conditions (T+I). T: TNF-α; I: IL-1β. (PDF) [file pgen.1004711.s007.pdf]

Table S3. Increased respiratory chain activities in skeletal muscle of the patient and similar respiratory chain activities in myoblasts of the patient and control, in basal and pro-inflammatory conditions. T+I : TNF-α + IL-1β.

**Frozen skeletal muscle**	**Control**		**Patient**	
**Spectrophotometric activities (nmol/min/mg proteins)**				
Complex I	17±4		56	
Complex II	35±7		105	
Complex III	300±56		600	
Complex IV	150±30		300	
Citrate synthase	96±18		228	
Lactate dehydrogenase	1758±415		3800	
**Myoblasts**	**Control**		**Patient**	
**Spectrophotometric activities (nmol/min/mg proteins)**	**Base**	**T+I**	**Base**	**T+I**
Complex I	13.8	11.4	10.5	10.4
Complex II	15.5	13.7	11.9	11.2
Complex III	168.4	176.7	156.8	147.1
Complex IV	46.3	42.8	35.7	35.3
Citrate synthase	51.8	51.2	44.6	44.8
Lactate dehydrogenase	9276.7	9875.3	8538.3	8882.7
