# Supplementary material for: A Thermolabile Aldolase A Mutant Causes Fever-Induced Recurrent Rhabdomyolysis without Hemolytic Anemia
Source: PLoS Genet. 2014 Nov 13;10(11):e1004711. doi: 10.1371/journal.pgen.1004711 (PMC4230727; doi:10.1371/journal.pgen.1004711)
Supplement: Table S4 — Normal fatty acid oxidation in patient myoblasts compared to control, in basal and pro-inflammatory conditions (TNF-α + IL-1β). (PDF) [file pgen.1004711.s008.pdf]

Table S4. Normal fatty acid oxidation in patient myoblasts compared to control, in basal and pro-inflammatory conditions (TNF-α + IL-1β).

	Patient		Control	
	**Basal conditions**	**Pro-inflammatory conditions**	**Basal conditions**	**Pro-inflammatory conditions**
**200 nmol of substrate**				
C4d5 Butyril- / IsobutyrilCn	564	631	579	570
C6d5 HexanoylCn	59	35	90	87
C8d5 OctanoylCn / ValproylCn	23	30	128	117
C10d5 DecanoylCn	85	63	295	239
C12d5 LauroylCn	133	109	214	185
C14d5 MyristoylCn	194	168	210	197
C16d5 PalmitoylCn	678	889	723	780
**50 nmol of subtrate**				
C4d5 Butyril- / IsobutyrilCn	481	479	326	401
C6d5 HexanoylCn	32	36	55	65
C8d5 OctanoylCn / ValproylCn	25	23	92	88
C10d5 DecanoylCn	41	47	159	180
C12d5 LauroylCn	53	38	49	68
C14d5 MyristoylCn	49	27	21	29
C16d5 PalmitoylCn	205	114	104	119
